# Longitudinal clinical trial enrollment trends across 341 US FDA-approved drugs and their guiding role in precision medicine strategies

**DOI:** 10.1038/s43856-025-01270-2

**Published:** 2025-12-05

**Authors:** Sophie Zaaijer, Simon C. Groen

**Affiliations:** 1 Division of Hematology and Oncology, Department of Medicine, University of California Irvine School of Medicine, Irvine, CA USA; 2 Cornell Tech, New York, NY USA; 3 Institute for Integrative Genome Biology, Department of Nematology, University of California Riverside, Riverside, CA USA

**Keywords:** Clinical trials, Drug development

## Abstract

**Background:**

Healthcare continues to suffer from a one-size-fits-all, trial-and-error model. Precision medicine seeks individualized care but requires clinical trial cohorts that mirror patient populations to detect differences in treatment response. Systematic assessments of trial cohort representativeness remain sparse; most studies are narrow. This study evaluates whether recent efforts, including the US FDA’s Drug Trial Snapshots Program, have improved demographic representation in pivotal trials across disease areas.

**Methods:**

We analyzed Drug Trial Snapshots Program data covering 341 Phase III clinical trials supporting drug approvals between 2017 and 2023. We compared enrollment of Asian, Black, Hispanic, and White participants with US Census data using chi-squared tests; assessed trends with regression; and examined patterns by disease area, regulatory designation, and US recruitment.

**Results:**

Here, we show that only 6% of pivotal clinical trials achieve enrollment aligned with the distribution of the four largest racial and ethnic groups in the US population. Enrollment of Black and Hispanic participants decreases over time, whereas that of Asian and White participants increases and remains stable, respectively. Trials based in the US include more Black participants, while trials with Breakthrough Therapy designation (which expedites development and review of drugs for serious or life-threatening conditions) show more balanced enrollment across groups.

**Conclusions:**

Persistent imbalances in trial enrollment limit the delivery of precision medicine. Trial location and regulatory designations influence who is included. Earlier planning of trials and more strategic site selection improve the potential for future treatments to serve individuals from all racial and ethnic groups.

## Abstract (web summary)

Zaaijer and Groen assess enrollment demographics across 341 FDA-submitted pivotal trials (2017–2023) supporting 348 approved drugs. They report persistent underrepresentation of Black and Hispanic individuals—especially in cancer and rare disease trials—alongside higher Black enrollment when trials are US-based and rising Asian participation.

## Plain language summary

Precision medicine tailors treatments to individuals. Therefore, trials for new therapies should enroll participants who reflect the populations that will use them. We analyzed enrollment in 341 trials supporting US Food and Drug Administration (FDA) approvals from 2017–2023. Black and Hispanic people were often underrepresented, while Asian and White people were better represented. This imbalance limits evaluation of genetic differences that may affect drug response. Improvements were seen in US-based trials and in those with Breakthrough Therapy designation, an expedited FDA pathway for serious conditions. We recommend practical steps: set representation targets early, use US-based sites when appropriate, and leverage regulatory tools. These measures support safer, more effective treatments for all communities and move health care toward precision medicine.

## Introduction

The US continue to grapple with cases of decreased life- and healthspan and billions of dollars in wasted expenses due to unanticipated side effects and low efficacies of certain drugs in subsets of individuals^1–3^. This coincides with a worsening strain on an overworked healthcare system and a continued rise in costs of healthcare insurance^4^. A key driver is the reliance on medical treatments that still follow a one-size-fits-all, trial-and-error model based on population averages that lack individualized, genetically informed prescribing models^5,6^.

Advancing precision medicine improves healthcare outcomes, directly addressing individual-specific genetic contributors to disease origin and drug response. To achieve the goals of precision medicine, ensuring adequate representation of different racial and ethnic groups in clinical trials is a crucial first step. In 2014, the US Food and Drug Administration (FDA) initiated the Drug Trial Snapshots Program (DTSP) to enhance transparency regarding representation of demographic groups in clinical trials that supported drug approvals for the US market, recognizing that recording and reporting participant ancestry in pivotal trial cohorts promotes awareness of potential enrollment biases in investigational drug testing^7^. A decade since its inception, we evaluate the DTSP’s impact and extract patterns from the FDA’s clinical trial enrollment data, which in turn may inform strategies for the future.

At the core of our investigation lies the acknowledgement that adequate representation of racial and ethnic groups in clinical trials is critical to the progress of implementing precision medicine at a larger scale. Many genetic variants of potential clinical relevance are found in all or most demographic groups but occur in highly variable frequencies across individual groups. Enrolling trial cohorts that are broadly representative of different racial and ethnic groups is therefore critical for increasing the likelihood that effects of genetic variants on drug efficacy and safety can be accounted for. For instance, inter-individual genetic variation in the cytochrome P450 monooxygenase (CYP) gene family can significantly influence drug metabolism across individuals. CYP enzymes metabolize 60-80% of drugs used in the clinic and CYP3A4 alone is involved in metabolizing 30-50% of these drugs^8,9^. As an example, the CYP3A4 variant rs274057 interacts with ≥22 drugs^10^. Remarkably, 76.5% of individuals with African ancestry carry a C allele at this locus versus only 2.78% of individuals with European ancestry. The CC genotype is associated with slower drug metabolism, resulting in reduced levels of certain active compounds in an individual’s circulatory system at a particular dose, which affects expected drug efficacy. Indeed, for treatment of breast cancer with cyclophosphamide, individuals carrying poor metabolizer CC alleles show lower survival rates^11^, which is most frequently observed among Black women^12^. Conversely, CC carriers have decreased odds for Opioid Use Disorder^13^. Therefore, trials composed predominantly of White participants would risk missing this allele, along with associated pharmacokinetic variations and clinically relevant phenotypes. Such unseen variations may result in unanticipated inefficacies of treatments or adverse outcomes for individuals carrying specific genetic variants, *regardless* of their ancestries.

Here, we analyze enrollment data from FDA-submitted pivotal clinical trials as a proxy for evaluating progress toward the goals of precision medicine. These analyses inform *short-term* strategies that may increase the likelihood of developing more equitable and effective treatments for individuals from all demographic groups in the *long term*. The dataset we have access to encompasses both US-based trials and those conducted internationally.

## Methods

This analysis follows Preferred Reporting Items for Systematic reviews and Meta-Analyses (PRISMA) guidelines (see checklist in Supplementary Data 1).

### Eligibility criteria

**Fig. 1:**
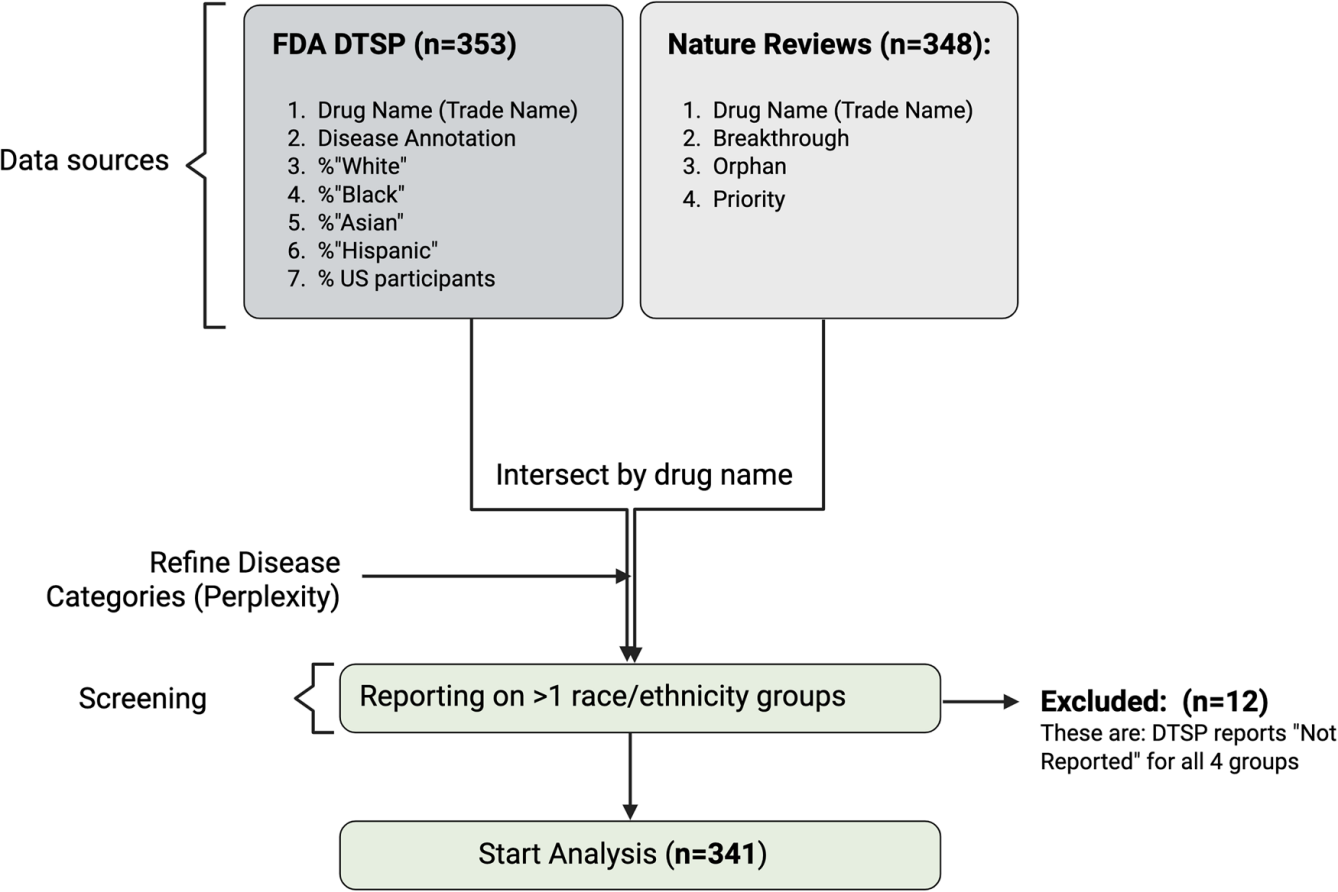
Flow diagram for Preferred Reporting Items for Systematic reviews and Meta-analyses (PRISMA). A total of 353 Drug Trial Snapshot Program (DTSP) reports for Phase III clinical trials that supported 348 US FDA Center for Drug Evaluation and Research (CDER) drug treatment approvals from 2017 to 2023 were identified. After excluding 12 DTSP reports because proportions of participants from different racial and ethnic groups were not provided, trial data and drug metadata from Nature Reviews Drug Discovery for 341 drug treatments were included in the data analyses.

All publicly available data from the FDA’s DTSP, which covers New Molecular Entities (NMEs) and Biologics License Applications (BLAs) approved between 2017 and 2023, were eligible for inclusion. The DTSP reports encompass 353 Phase III clinical trials that supported the 348 FDA Center for Drug Evaluation and Research (CDER) approvals in this time period (2017, *n* = 46^14^; 2018, *n* = 59^15^; 2019, *n* = 48^16^; 2020, *n* = 53^17^; 2021, *n* = 50^18^; 2022, *n* = 37^19^; 2023, *n* = 55^20^; Fig. 1; Supplementary Data 2).

Five drug approvals included two separate clinical trial demographic datasets (Rydapt, Nuzyra, Zegalogue, Vabysmo, and Relyvrio), explaining the numerical difference between the DTSP data and the Literature (Fig. 1; Supplementary Data 2).

A total of 12 trials were excluded due to lack of any demographic reporting; none were excluded for reporting participation of only one of the racial and ethnic groups included in the DTSP reports (“Asian,” “Black,” “Hispanic,” and “White,” see below; Fig. 1; Supplementary Data 2).

### Race and ethnicity annotation

To evaluate representation of racial and ethnic groups in pivotal clinical trials, we adopted the demographic categories used in the US FDA’s DTSP reports. Specifically, we analyzed four categories or groups—Black or African American, Asian, White, and Hispanic or Latino—following the framework outlined in the Office of Management and Budget (OMB) Statistical Policy Directive No. 15 (SPD-15), which provides federal standards for race and ethnicity reporting^21^.

While SPD-15 defines additional categories, including American Indian or Alaska Native and Native Hawaiian or Other Pacific Islander, these groups are excluded by the FDA because of consistently low enrollment across trials, precluding statistically meaningful comparisons with these demographic groups.

It is important to note that these annotations are self-reported by trial participants and not verified genetically: participants typically select their race or ethnicity from predefined categories at enrollment. While these labels provide a broad overview of demographic group representation in clinical trials, they are imprecise proxies for genetic ancestry and fail to capture important within-group variation (e.g., East Asian versus South Asian).

Our methodology mirrors the FDA’s DTSP approach and aligns with current regulatory standards while ensuring the dataset remains interpretable and statistically robust.

### Drug Trial Snapshots Program report data extractions and limitations

Column headers and annotations in the DTSP reports were standardized across years to address variability in reporting practices. The reports contain data that is not always disaggregated and, in certain categories, data is missing for some of the years covered by our analyses:

- Demographic data specific to US trial sites are not disclosed; only aggregated overall participant demographics are available.
- The proportion of trial activity conducted within the US is given for 2017, 2018, 2019, 2020, and 2023 but is missing for 2021 and 2022.
- The FDA did not start with reporting on individual trial sizes until 2022.

#### Screening and data extraction

The DTSP reports provide demographic breakdowns that reflect the overall composition of each clinical trial cohort and are used by the FDA to assess efficacy and safety outcomes. The FDA notes that DTSP data are limited to the information available at the time of the original regulatory approval^22^.

#### Disease classification

The FDA did not annotate disease categories for 27% (*n* = 94) of the drugs approved between 2017 and 2023. Furthermore, the disease classifications that were provided lacked consistency across years—for example, “Heart Blood Kidney Endocrine Diseases” in 2021 versus “Hematology” in 2019. To enable uniform classification, the Artificial Intelligence tool *Perplexity* was used to assign a disease category to each approved treatment indication. The accuracy of *Perplexity*’s classifications was validated through cross-referencing with available FDA annotations, demonstrating a 98% concordance (*n* = 345; Supplementary Data 2).

Information on FDA’s drug designations “Orphan,” “Breakthrough Therapy” and “Priority Review” for all years were retrieved through literature review: 2017^14^; 2018^15^; 2019^16^; 2020^17^; 2021^18^; 2022^19^; 2023^20^ (Fig. 1; Supplementary Data 2).

### Data synthesis

#### Longitudinal analysis of representation

For each year between 2017 and 2023, the number of pivotal clinical trials was counted in which the representation of a racial or ethnic group was not significantly lower than the group’s representation in the US Census population (as determined by conducting two-sided χ²-tests with a significance threshold of *P* < 0.05 in GraphPad Prism). If the representation percentage of a demographic group in a clinical trial cohort is smaller than the group’s US Census representation percentage, then this suggests that the group was not sufficiently represented in a trial when combined with a significant χ²-test outcome.

The number of trials in which a demographic group was deemed sufficiently represented was summed per year, and the sums for each year were used as input for regression analysis to detect longitudinal trends in representation for each group in clinical trials. Linear and quadratic regressions were performed for each demographic group for the period 2017–2023, and the regression with the highest F-value was used to infer the longitudinal pattern of representation in clinical trials per group. Regressions were performed using GraphPad Prism. F-tests were two-sided and used a significance threshold of *P* < 0.05.

The same analyses were repeated after subdividing the clinical trial dataset according to drugs’ designations as “Orphan”, “Priority Review”, and “Breakthrough Therapy” drugs to determine potential effects of these designations on longitudinal patterns of representation for each demographic group. Differences in distributions across subdivided datasets were analyzed with two-sided Kolmogorov-Smirnov tests and used a significance threshold of *P* < 0.05.

Finally, considering all pivotal trials reported by the DTSP from 2017 to 2023, the number of trials was tallied that showed sufficient representation of each racial and ethnic group alone and in all possible combinations by building a Venn diagram.

#### Analysis of representation by disease category

Representation percentages of each individual demographic group per trial for drugs approved in the years 2017-2023 were analyzed across disease categories using one-way ANOVA followed by post-hoc Tukey’s HSD tests in GraphPad Prism. F-tests were two-sided and used a significance threshold of *P* < 0.05.

#### Analysis of representation by trial location

Correlation analyses between percentages of US-based clinical trial participants and representation percentages of each individual demographic group per trial for drugs approved in the years 2017-2023 were performed using linear regression in GraphPad Prism. F-tests were two-sided and used a significance threshold of *P* < 0.05.

## Results

### Reporting transparency

Using the four largest federally recognized race and ethnicity categories within the US—Asian, Black, Hispanic, and White—as defined by OMB Directive 15 and adopted in the FDA’s DTSP reports (“Methods”), we evaluate enrollment patterns in 341 pivotal trials that led to treatments approved by the FDA for the US market from 2017 to 2023 (Fig. 1). We find that race and ethnicity of trial participants are reported relatively consistently for White (100%), Black (96%), and Asian (95%) individuals but less so for Hispanic (90%) individuals (Supplementary Data 3). The DTSP reports for the majority of trials provide enrollment information for all four demographic groups (85%, *n* = 288), followed by reports with information for three groups (13%, *n* = 43), two groups (3%, *n* = 9) or one (0.3%, *n* = 1) (Supplementary Data 4**)**.

### Representation of demographic groups in trials of drugs approved in 2023 for the US market

**Fig. 2:**
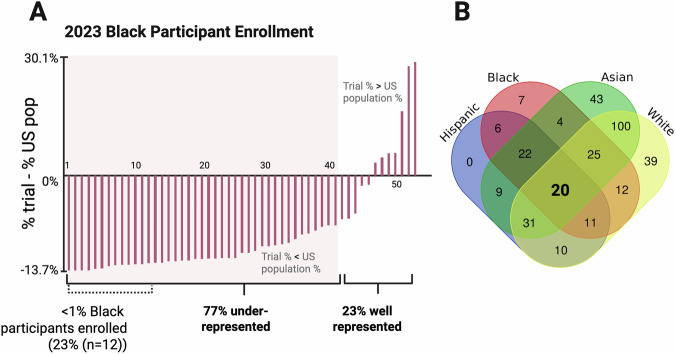
Enrollment patters for clinical trial participants from different racial and ethnic groups. **A** Representation of Black individuals in each pivotal trial that led to a FDA-approved drug in 2023. X-axis: Each red column represents a trial. For 53 of the 55 drug trials the percent enrollment of Black individuals was reported by the DTSP. Y-axis: The percent enrollment of Black individuals is subtracted from their US Census statistics for 2023 (13.7%). Clinical trials inside the light red box show significant underrepresentation of Black individuals (two-sided χ^2^ tests, *P* < 0.05; Supplementary Data 5). **B** Venn Diagram depicting the number of pivotal clinical trials for drugs approved in the 2017-2023 period that represent one or more human population groups appropriately (two-sided χ^2^ tests, *P* ≥ 0.05; Supplementary Data 5). The number of trials that represented all four demographic groups well is indicated in bold.

Among the 55 pivotal trials for drugs approved in 2023 we observe that only 23% of trials (*n* = 12) had representative numbers of Black participants (Fig. 2a), and just 30% of trials (*n* = 16) had enrollment representative for Hispanic individuals (Supplementary Fig. 1a) relative to US Census data. On the other hand, Asian individuals were represented appropriately in 81% of trials (*n* = 44; Supplementary Fig. 1b), surpassing the proportion of trials in which White individuals were well-represented (Supplementary Data 5).

### Major variability in representation among individual drug trials

Considering all pivotal trials for drugs approved from 2017 to 2023, out of 341 trials only 6% (*n* = 20) represented *all* four major demographic groups sufficiently. In 26% of trials (*n* = 89) three groups were represented appropriately and in 41% (*n* = 141) two groups. In 26% of trials (*n* = 89) only a single group was represented well and 0.6% (*n* = 2) did not represent any group according to the FDA-stated guidelines (Fig. 2b).

### Mixed longitudinal trends in trial representation of demographic groups for drugs approved since 2017

**Fig. 3:**
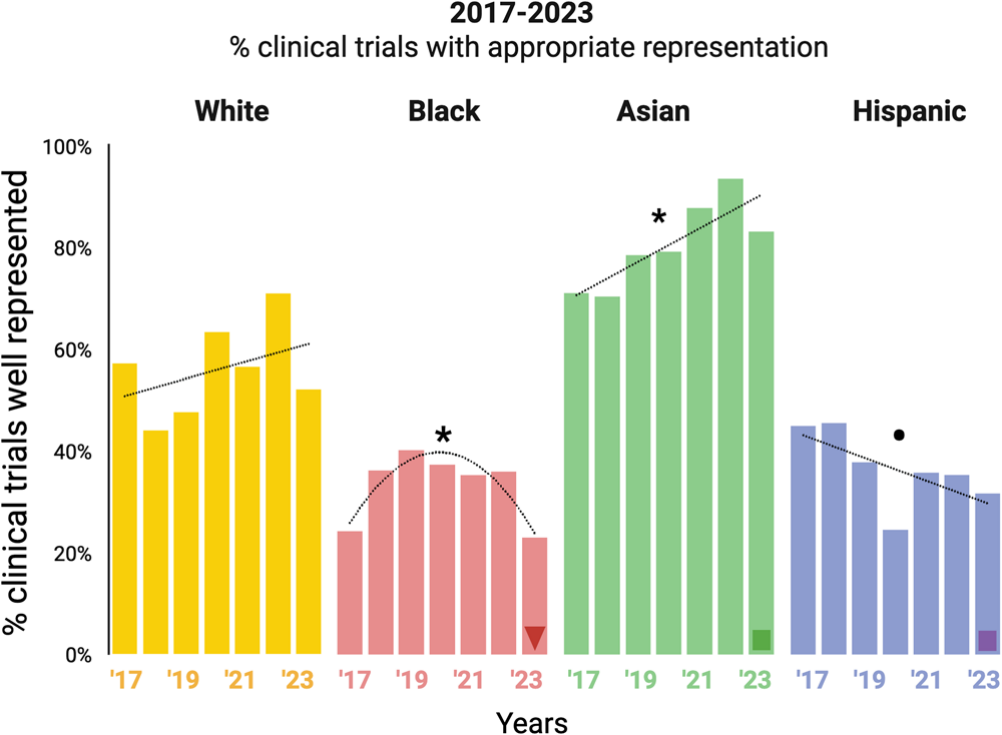
Representation of racial and ethnic groups in clinical trials over time as reported by the DTSP. X-axis: All trials for each year are represented in a single column, clustered by human population group. Y-axis: The percent clinical trials that show appropriate representation of a group (two-sided χ^2^ tests, *P* ≥ 0.05; Supplementary Data 5) compared to the US Census data for that year. The dotted lines indicate the best fitting linear or quadratic regression as determined by two-sided F-tests and two-sided χ^2^ tests (Black: F_quadratic_ = 13.36, *P* = 0.017; Asian: F_linear_ = 12.00, *P* = 0.018 and χ^2^_2017-2023_ = 7.862, *P* = 0.0050; Hispanic F_linear_ = 3.85, *P* = 0.1071 and χ^2^_2017-2023_ = 7.901, *P* = 0.0049; * indicates *P* < 0.05 and • indicates *P* ≤ 0.1). The data underlying the column for Black individuals in 2023 (triangle) is shown in Fig. 1a, and underlying data for Asian (green square) and Hispanic (purple square) individuals is shown in Supplementary Fig. 1.

Analysis of longitudinal patterns of representation across individual trials over the period 2017–2023 uncovers that while representation of White individuals does not change significantly with time, representation of Asian individuals increases (F_linear_=12.00, *P* = 0.018; χ^2^_2017-2023_ = 7.862, *P* = 0.0050; Fig. 3). However, we observe a decline in representation for Black individuals since a peak in 2019 (F_quadratic_=13.36, *P* = 0.017) and a gradual decline for Hispanic individuals since 2017, including a stark decrease in 2020 (F_linear_=3.85, *P* = 0.1071; χ^2^_2017-2023_ = 7.901, *P* = 0.0049; Fig. 3).

### Trials for rare diseases systematically lack appropriate representation

**Fig. 4:**
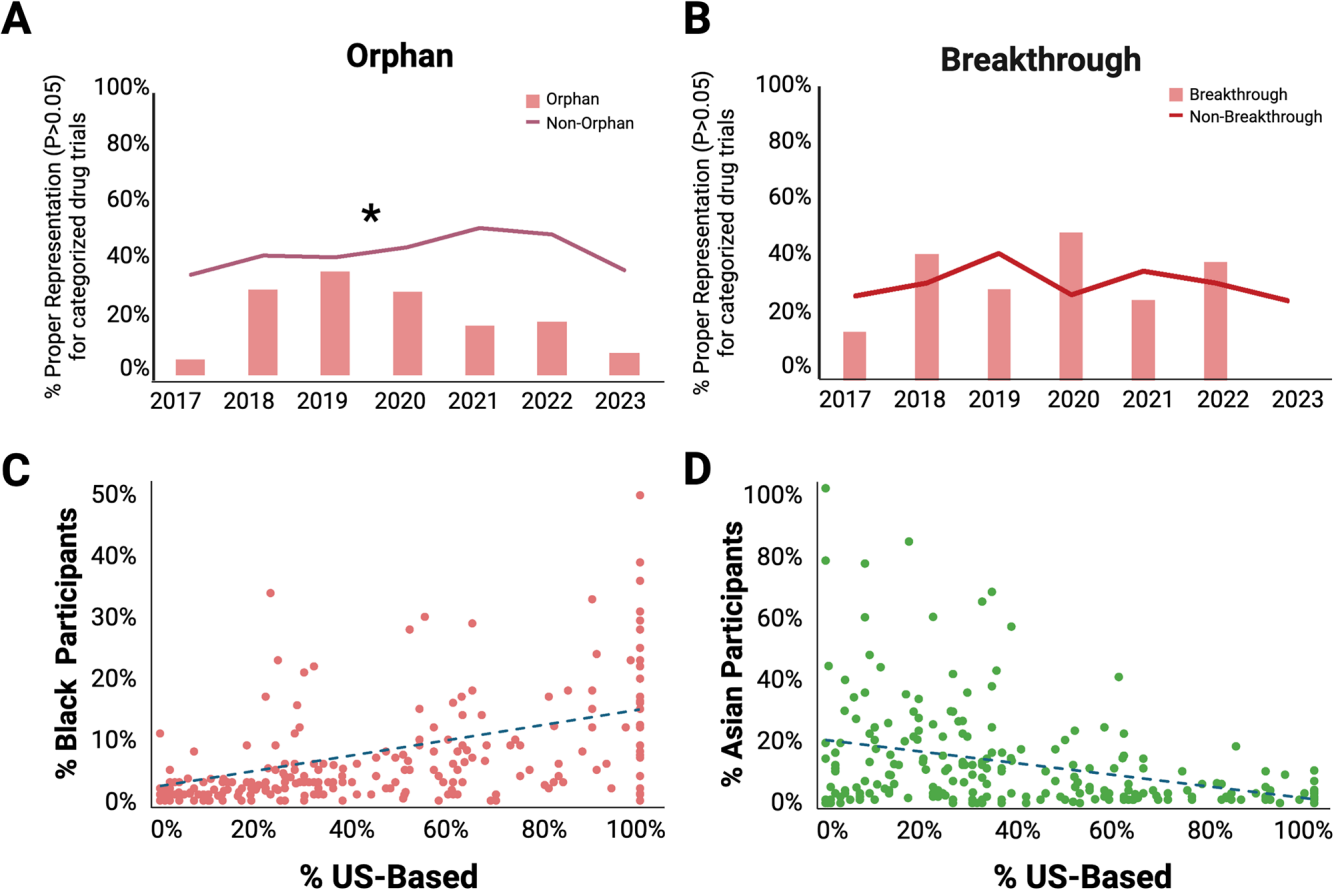
Representation of racial and ethnic groups in clinical trials over time in relation to drug designation and trial location. **A** Representation of Black individuals in trials for Orphan and Non-Orphan drugs. X-axis: All trials for Orphan drugs for each year are represented in a single column, while all trials for Non-Orphan drugs for each year are represented in a data point on the line. Y-axis: The percent trials that show appropriate representation of Black individuals (two-sided χ^2^ tests, *P* ≥ 0.05; Supplementary Data 5) compared to the US Census data for that year. The asterisk indicates a significant difference in representation between trials for Orphan and Non-Orphan drugs across years (Kolmogorov-Smirnov test; KS D_Black_ = 0.86, *P* = 0.008). **B** The same analyses as in (**A**) were repeated after separating clinical trials by those for drugs with and without Breakthrough Therapy designation. **C** Regression analysis of representation of Black individuals in trials on the proportions of trial participants based in the US. X-axis: Each dot represents a trial. Y-axis: The percent enrollment of Black individuals in each trial is shown. The dotted line indicates a significant linear regression fit as determined by a two-sided F-test (*F* = 25.12, *P* < 0.0001). **D** The same analysis as in (**C**) was repeated for representation of Asian individuals (two-sided F-test; *F* = 40.33, *P* < 0.0001).

In 2023, 28 of the 55 approved drugs received Orphan designation, assigned for drugs that treat rare diseases (occurring in ≤65 per 100,000 individuals). Longitudinal analysis from 2017 to 2023 shows consistently lower representation of Black and Hispanic individuals in pivotal trials for Orphan drugs compared to Non-Orphan drugs (KS D_Black_ = 0.86, *P* = 0.008; KS D_Hispanic_ = 1.00, *P* = 0.000058; Fig. 4a, Supplementary Fig. 2), whereas representation of Asian and White individuals shows no differences (Supplementary Fig. 2).

### Relationship between drug designation and representation of demographic groups

Similar to pivotal trials for drugs with Orphan designation, trials for drugs with Priority designation demonstrate a significant difference in representation of Black or Hispanic individuals (Supplementary Fig. 3). In contrast, Breakthrough designation—an accelerated FDA pathway for serious conditions backed by preliminary evidence of substantial benefit—leads to an improvement in representation of Hispanic individuals, with a significant difference observed for drugs with or without this designation (KS D_Hispanic_ = 0.86, *P* = 0.00483; Supplementary Fig. 4). Pivotal trials for drugs with Orphan and/or Priority designations make up over 72% (*n* = 246) of all 341 trials. Breakthrough designation was given to 29% (*n* = 98) of the trials but this designation is always combined with either the Priority and/or Orphan designations.

Interestingly, underrepresentation of Black individuals decreases from 71.4% of trials for Priority drugs (*n* = 85 of 119 trials) to 63.7% (*n* = 58 of 91 trials) with addition of the Breakthrough designation, although this is not statistically significant (χ^2^ = 2.9, *P* = 0.089, Supplementary Data 5). Furthermore, across trials for Orphan drugs, underrepresentation of Black individuals falls from 78.4% (*n* = 76 of 97 trials) to 63% (*n* = 46 of 73 trials) with the Breakthrough designation added (χ^2^ = 13.87, *P* = 0.0002, Supplementary Data 5). Conversely, underrepresentation of Hispanic individuals increases from 54.6% of trials for Priority drugs (*n* = 65 of 119 trials) to 69.2% (*n* = 63 of 91 trials) with addition of the Breakthrough designation (χ^2^ = 8.61; *P* = 0.0033). No significant difference is observed with regards to representation of Hispanic individuals in trials for Orphan drugs with or without Breakthrough designation.

### Geographic location of clinical trials influences representation of demographic groups

The proportion of US-based participants in trials positively correlates with enrollment of Black individuals (*F* = 25.12, *P* < 0.0001; Fig. 4c). For trials that were 100% US-based, the average representation of Black individuals was 17.7% (Range: 0-75%, Median: 15%). In contrast, an inverse trend is observed for representation of Asian individuals (*F* = 40.33, *P* < 0.0001; Fig. 4d). For trials that were 100% US-based, the average enrollment of Asian individuals was 2.3% (Range: 0-10.3%, Median: 2%). Correlation between representation of Hispanic individuals and the relative number of US-based trial participants is less pronounced but positive (*F* = 6.05, *P* = 0.0147; Supplementary Fig. 5).

Longitudinal patterns show that trials’ proportions of US-based participants increase from a median of 33% for drugs approved in 2017 to 47% in 2020. However, we observe a decline to 25% for those approved in 2023 (Supplementary Fig. 6, Supplementary Data 6).

We next ask if drug designation could drive the choice of location for pivotal trials. Trials for drugs with Orphan designation had fewer US-based participants compared to trials for Non-Orphan drugs (39% vs 47%; Student’s *t* = 2.0078, *P* = 0.0457), which is in keeping with the observed lower representation of Black individuals in pivotal trials for Orphan drugs (Supplementary Fig. 7).

No significant relationship is found between Priority or Breakthrough designations and the proportion of US-based trial participants when evaluating averages (Supplementary Fig. 7). However, when we analyze the variance in the percentages of US-based participants, trials for Breakthrough drugs show a bias for more robust US-based localization across individual trials (Levene’s test, *P* = 0.003685). Trials for drugs without Breakthrough designation were either minimally based in the US (10–20% of participants) or for the majority based in the US (80-100%). The higher consistency in the levels of US-based participants (20-40%) in trials for drugs with Breakthrough designation is in agreement with the equal representation of Black individuals in trials for drugs with and without this designation (Supplementary Fig. 7).

### Bias in representation of demographic groups for specific disease types

**Fig. 5:**
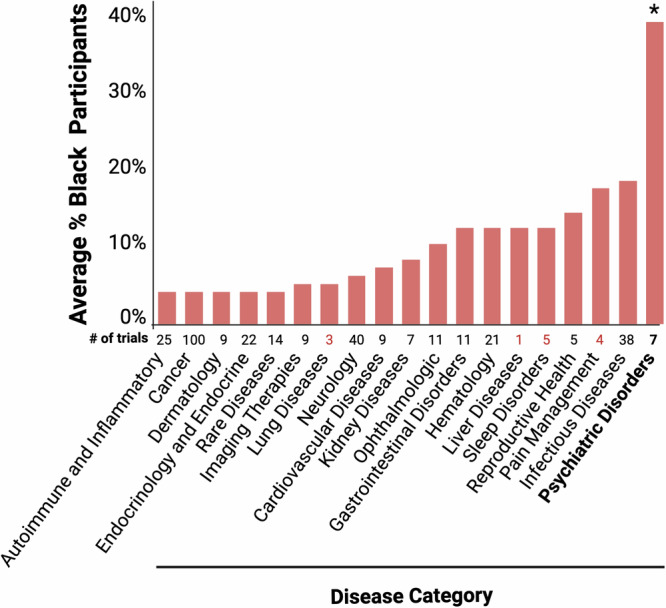
Representation of Black individuals in clinical trials for drugs approved between 2017 and 2023 separated by disease category. X-axis: All trials for each disease category are represented in a single column. Y-axis: The percent enrollment of Black individuals across trials for each disease category is shown. The disease category Psychiatric Disorders is marked with an asterisk since the level of representation of Black individuals in trials for that category (average of 38% in *n* = 7 trials) is significantly higher than their level of representation in trials for the majority of other disease categories, with the exception of Infectious Diseases, Reproductive Health, and Sleep Disorders (one-way ANOVA with post hoc Tukey’s HSD tests, P≤0.04569).

Over the period of 2017-2023 we observe significantly higher enrollment of Black individuals in clinical trials that test drugs for Psychiatric Disorders relative to most other disease types and these trials were 89% US-based (Fig. 5). Trials for Pain management (*n* = 4) show a 17% enrollment of Black participants and were 100% US-based. Trials for Infectious Diseases form an exception to this pattern with 18% and 20% enrollment of Black and Hispanic participants, respectively, even though these trials were only 35% US-based (Supplementary Data 7).

Drug trials for Cancer are among the lowest on average for representation of Black individuals (4% over *n* = 100, 40% US-based) together with trials for Dermatology (4% over *n* = 9, 61% US-based), Endocrinology (4% over *n* = 22, 32% US-based), and (as previously confirmed) Rare Diseases (Fig. 5). Trials for Psychiatric Disorders enrolled the lowest percentage of White (55%) and Asian (2%) participants but show a 20% enrollment of Hispanic individuals (Supplementary Fig. 8).

Asian individuals were on average most highly represented in drug trials for Autoimmune Diseases (17% over *n* = 25, 23% US-based), Kidney Diseases (15% over *n* = 15, 17% US-based), and Cancer (14% over *n* = 100, 40% US-based), while Hispanic individuals were on average most highly represented in drug trials for Gastrointestinal Disorders (23% over *n* = 11, 59% US-based), Reproductive Health (21% over *n* = 5, 75% US-based), and Dermatology (20% over *n* = 9, 61% US-based; Supplementary Data 7).

## Discussion

In this study, we evaluated decadal trends in representation of racial and ethnic groups in clinical trials to understand the progress made towards implementation of precision medicine and identify strategies for further improvement.

Enrollment of participants from different demographic groups varied by disease area and was linked to the proportion of US-based trial participants. It is unclear why (independent) clinical trial sponsors that work on one disease type, such as Psychiatric Disorders, choose to perform their trials mostly in the US and (therefore) are able to recruit appropriate numbers of both Black and Hispanic participants, while sponsors of Cancer trials choose differently.

The low representation of Black individuals in trials for Cancer treatments is a concern. Cancer is a disease group where Black individuals experience consistently higher mortality rates^23^. Numerous confounding variables are at play, such as delays in diagnosis or access to health care. A number of companies have started campaigns to address these issues, as described in DEI reports^24^. This is a crucial step forward, and results of these efforts will emerge in the years to come.

Trials for treatments of Infectious Diseases form an exception, with higher enrollment of Black and Hispanic participants, even though fewer US-based participants are recruited for these trials. This was expected, since many infectious diseases, such as infections with parasitic nematodes that may be treated with anthelmintics, have higher prevalence in Africa and Latin America^25^.

We find a correlation between representation of Black individuals and the extent to which trials are based in the US. The data suggests that merely hosting clinical trials in the US increases the chance of representing this demographic group according to its contribution to the US Census population. These statistics align with those of previous analyses performed by the FDA itself, which demonstrated that Black individuals are on average represented well in clinical trials performed in the US^26^. However, when the FDA reviews a novel treatment for regulatory approval in the US, it relies not only on data generated in US-based trials but also on data from international trials. Therefore, solely focusing on improving the representation of racial and ethnic groups in US-based trials is not sufficient.

Addressing disparities in representation of racial and ethnic groups within pivotal trials that support FDA approvals of novel treatments may require reviewing clinical trial design through a global lens—one that prioritizes regulatory alignment across jurisdictions. To file for regulatory approval, pharmaceutical companies currently use a single global Phase III trial, with data submitted simultaneously to multiple regulatory agencies, which accelerates access to international markets.

This approach is enabled by harmonized regulatory frameworks established by the International Council for Harmonization (ICH), comprising bodies such as the FDA (US), EMA (EU), PMDA (Japan), and others. Under the ICH framework, mutual recognition of trial data is permitted, provided studies adhere to Good Clinical Practice (GCP) guidelines.

As of 2024, 17 regulatory authorities are full ICH members, meaning they both adhere to *and* enforce ICH standards. Pharmaceutical companies favor these countries for trial site selection because of reduced complexity regarding compliance and greater regulatory predictability. Indeed, according to IQVIA, the US accounts for 16% of global trial site usage, followed by China (13%), and Western European countries (25% cumulatively), which are all ICH members.

In contrast, 26 countries are observers, but not members, of ICH, such as India and South Africa. While observers adopt ICH principles, trial data collected in these countries may face greater scrutiny and require additional documentation to meet approval standards. Averse to regulatory uncertainties and higher perceived risk, pharmaceutical companies therefore organize less than 3% of global trials in non-member states, including countries in sub-Saharan Africa and Southeast Asia^27–29^.

Of the ICH member countries, the US has the population with possibly the highest percentage of individuals with African ancestry. This aligns again with our observation that US-based trials have higher representation of Black individuals.

For Hispanic individuals, a promising shift occurred recently when Brazil joined the ICH as a full member in 2016^30^, Mexico in 2021^31^, and Argentina in 2024^32^. These milestones may help increased trial activity in Latin America, enhancing representation of Hispanic individuals through clinical development programs in the years to come.

The proportion of US-based participants in pivotal trials rose to a peak of 47% in 2020, before declining to a median of 25% by 2023. Given the clear association between representation of Black individuals and the proportion of US-based trial participants, the decline in appropriate trial enrollment of Black participants between 2021 and 2023 likely reflects this geographic shift.

Following China’s accession to ICH in 2017, its clinical trial activity expanded rapidly; by 2023, China had doubled its global share and emerged as a major hub for both academic and commercial research. This growth also follows the trend in representation of Asian individuals across trials.

Although the US has made measurable progress toward more inclusive recruitment, the global nature of pivotal trials continues to skew collective enrollment toward formation of cohorts predominantly consisting of White individuals. Supporting countries that have relatively high numbers of Black or Hispanic individuals in their populations with meeting ICH standards—and facilitating their eventual membership—could help address this gap.

A Breakthrough Therapy designation for a novel treatment facilitates earlier and more iterative engagement of trial sponsors with the FDA during the drug development process. By contrast, a Priority Review designation accelerates regulatory evaluation but does not influence clinical trial design and conduct.

Trials granted Priority Review without receiving an additional Breakthrough Therapy designation demonstrated lower recruitment levels of Black individuals. These drugs were more likely to undergo post-marketing label revisions than those reviewed under standard timelines^3,33^.

Conversely, adding the Breakthrough Therapy designation increased representation of Black individuals in clinical trials for a large number of drugs. Importantly, this designation appears to be associated with an additional advantage: Breakthrough therapies have been reported not to show an increase in post-marketing adverse effects compared to drugs without this designation^34^, suggesting that expedited development does neither compromise safety nor engender additional costs for having to organize Phase IV trials. Altogether, these findings imply that pharmaceutical companies more systematically focus on US-based trial sites when a drug receives Breakthrough Therapy designation, benefitting from the US’ alignment with ICH guidelines, predictable US health operational standards, and short lines of communication between trial sites and FDA officers^35,36^. The findings further suggest that receiving this designation nudges trial sponsors to plan participant recruitment earlier and more carefully, resulting in trial cohorts with a more balanced demographic make-up.

Enrollment of Hispanic participants in clinical trials remains stubbornly low, both in the US^9^ and globally, with few signs of improvement over the past decade. Recruitment of Black participants seems to have received greater attention, reflected in policy initiatives and reporting practices. For example, the DTSP began reporting on enrollment of Hispanic individuals only in 2017, whereas representation of Asian, Black, and White individuals has been tracked since 2015. A PubMed search yields 16,380 articles on ‘Black’ and ‘clinical trial’ but only 6264 on ‘Hispanic’ and ‘clinical trial,’ underscoring the disparity in focus and potentially explaining why trial recruitment of Hispanic participants has not followed the same trends as seen for Black participants. It also demonstrates that focused efforts can lead to improvements as seen for clinical trial representation of Black individuals.

Although the US Congress passed the National Institutes of Health Revitalization Act in 1993 to improve representation of racial and ethnic groups in clinical trials, it was not until the significant drop in DNA sequencing costs around 2010-2015 that more meaningful scientific discoveries became possible, as evidenced by a surge in PubMed publications and scientific insight^32^. This revolution also proved a juncture in our understanding that ancestral background of participants could be used as a helpful temporary proxy to pave the way towards true precision medicine^37^. Since clinical trials span 2-8 years and the trials reported in this study may have been initiated as early as 2009, these advances in scientific understanding will likely not yet have contributed to more balanced representation of racial and ethnic groups for drugs approved in 2017. However, given the fact that US-based clinical trial sites have increased from 19% in 2019 to 23% in 2023^38^, we expect appropriate representation of demographic groups will improve in the years to come.

Factors that could further drive this improvement are announcements by the pharmaceutical industry since 2020 to commit to representative clinical trials, as evidenced by their Diversity Commitment statements^33,34^. Additionally, a significant regulatory development is the FDA’s 2020 release of the draft guidance on Diversity Action Plans (DAPs), which recommends sponsors submit DAPs during the period between Investigational New Drug (IND) application and Phase III trial initiation. While not mandatory at the time, 91 sponsors voluntarily submitted DAPs during the 2020-2021 period (91 out of 1,159 Phase III trials started in 2020^39,40^). Encouragingly, 84% were submitted by oncology divisions.

Government guidance and support are crucial tools for the biomedical field. Our findings reveal that FDA-approved drugs had significantly higher frequencies of reporting demographic information for trial cohorts (82-100%) compared to ongoing clinical trials (43%^41^), underscoring the effectiveness of centralized government initiatives.

Recruiting cohorts of clinical trial participants that represent a wide range of ancestries helps to account for effects of alleles that occur at different frequencies across racial and ethnic groups, but current enrollment goals and strategies could be criticized for their degree of relevance towards advancing precision medicine. First, alignment to the US Census data is not necessarily germane to the demographic composition of the human population at a global scale (discussed above), nor does this alignment hold biological relevance. Regarding the latter point, allelic frequencies (particularly for genes affecting clinical outcomes) in the human population should ideally guide recruitment practices^42,43^. Second, current practices are limited by the imprecision of self-reported race and ethnicity^43^. These categories introduce ambiguity around individuals’ genetic ancestries, particularly in admixed populations or across borders (“Black” in the US may genetically mean something different than in other countries), and may misrepresent the distribution of clinically relevant genetic variants among the population. A more precise long-term strategy involves stratifying participants based on allelic profiles, bypassing self-reported race and ethnicity as a proxy. However, because many clinically relevant variants in the population are yet to be characterized, and genomic screening is not yet routinely implemented in clinical trials or care, this approach is currently not feasible at scale.

As deployment of genome sequencing, accurate clinical phenotyping, development of regulatory frameworks, improvement of data security, and building of trust continue to advance in the coming decades, deciding factors for trial recruitment are expected to shift from self-reported race and ethnicity to individualized genome-wide profiles of genetic variants under the umbrella of precision medicine. However, until universal genomic profiling becomes standard practice, trial recruitment based on self-reported race and ethnicity remains an excellent first step to increase the likelihood of being able to account in clinical trials for important alleles that segregate at different frequencies among demographic groups, leading to improved precision medicine pipelines.

Appropriate representation of racial and ethnic groups must inform all stages of drug development. However, after FDA approval, a reliance on race and ethnicity as treatment proxies can become problematic, since most traits are polygenic in nature and genetic variants are not private to certain demographic groups alone^44–46^. Patient cohort ancestral profiles can guide preclinical research^47^ and trial stratification, but only as a bridge toward biomarker-driven precision medicine in the clinic—ultimately enabling care for all individuals, regardless of their ancestry.

In this study, we used data from pivotal clinical trials in phase III for drugs that obtained FDA approval. A persistent challenge in the field is that data about failed trials *and* statistics on representation of racial and ethnic groups among trial cohorts are not frequently released. This data could uncover effects of the representation of demographic groups on the success of clinical trials and approval of new drug treatments. Such data could also identify core variables that need special attention during the design of trials in each consecutive phase of drug development and after FDA approval.

## Conclusion

Our analysis shows that participant enrollment trends across clinical trials remain troubling for Black and Hispanic individuals, while Asian and White individuals are better represented. Trial geography strongly influences patterns of participant enrollment: participation of Asian individuals is highest when studies are conducted outside the US, whereas that of Black individuals improves when trials are US-based. Regulatory pathways also matter—Breakthrough Therapy designation appears to influence choice of trial location, supporting better representation of Black individuals by encouraging early engagement of trial sponsors with the FDA and more deliberate trial design.

Despite a well-established vision for precision medicine, the earliest steps toward building clinical trial cohorts that are truly representative of the demographic make-up of the targeted patient population remain hindered by global enrollment practices that still cause cohorts to be demographically homogeneous all too often. While the US is making measurable progress, the international nature of where pivotal trials are conducted sustains a bias toward White- and Asian-majority populations. Expanding ICH membership to countries with higher proportions of Black and Hispanic individuals in their demographics could, over time, move development of clinical treatments closer to the inclusive foundation that precision medicine requires as an initial step.

## Supplementary information

Supplemental Information

Description of Additional Supplementary files

Supplementary Data 1

Supplementary Data 2

Supplementary Data 3

Supplementary data 4

Supplementary Data 5

Supplementary Data 6

Supplementary Data 7

## Data Availability

The data analyzed in this study are available in Supplementary Data 2 associated with this manuscript.
