# Supplementary material for: Longitudinal clinical trial enrollment trends across 341 US FDA-approved drugs and their guiding role in precision medicine strategies
Source: Commun Med (Lond). 2025 Dec 5;5:514. doi: 10.1038/s43856-025-01270-2 (PMC12680696; doi:10.1038/s43856-025-01270-2)
Supplement: Supplementary file 1 — Supplemental Information [file 43856_2025_1270_MOESM1_ESM.pdf]

SUPPLEMENTAL FIGURES

Longitudinal Clinical Trial Enrollment Trends across 341 US FDA-Approved Drugs and Their Guiding Role in Precision Medicine Strategies

Sophie Zaaijer,[a,b,c],* Simon C. Groen[c],*

[a]University of California Irvine, Irvine, CA, USA;
[b]Cornell Tech, New York, NY, USA;
[c]University of California Riverside, Riverside, CA, USA.

*Corresponding authors at: University of California Riverside, Genomics Building 2202A, Riverside, CA, 92521, USA.

E-mail addresses: sophie@cornell.edu (S. Zaaijer); simon.groen@ucr.edu (S.C. Groen).

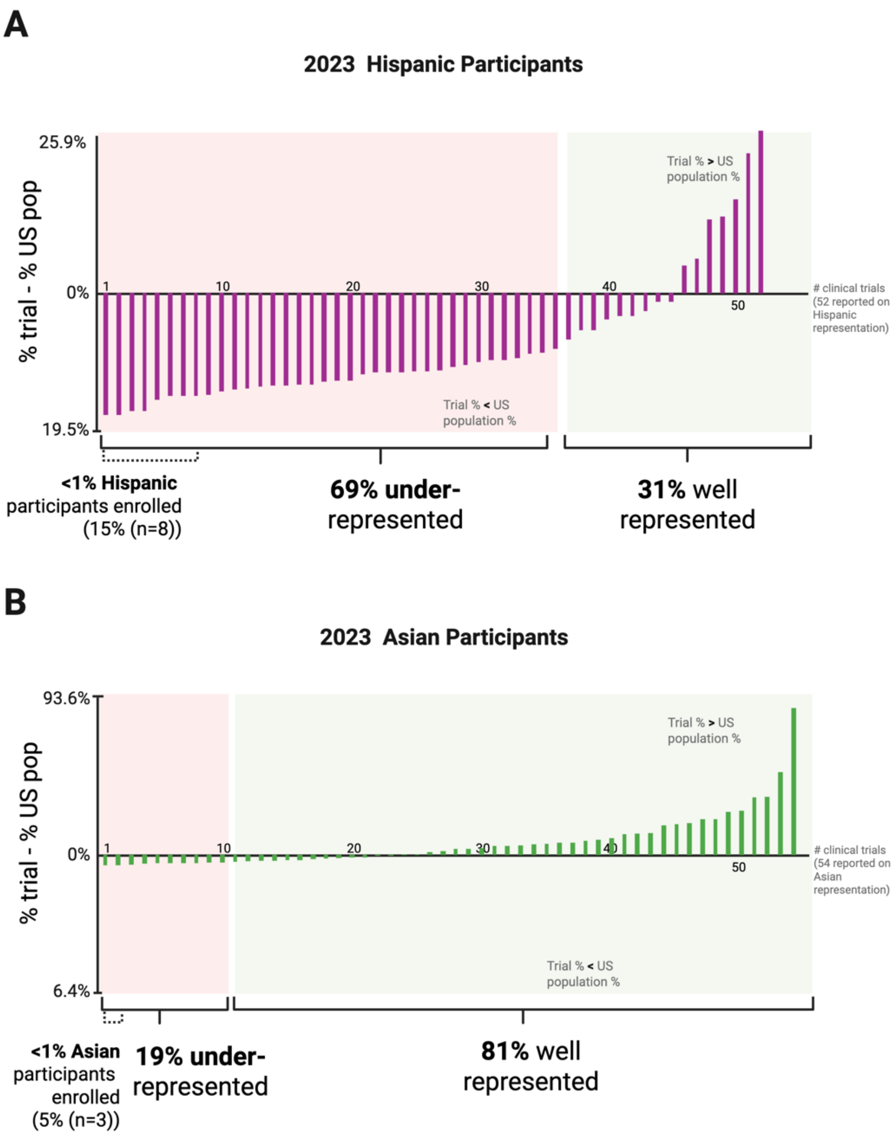

Supplementary Fig. 1 | Enrollment patters for clinical trial participants from different racial and ethnic groups. a. Representation of Hispanic individuals in each pivotal trial that led to a FDA approved drug in 2023. X-axis: Each red column represents a trial. For 52 of the 55 drug trials the percent enrollment of Hispanic individuals was reported by the DTSP. Y-axis: The percent enrollment of Hispanic individuals is subtracted from their US Census statistics for 2023 (19.5%). Clinical trials inside the light red box show significant underrepresentation of Hispanic individuals (two-sided χ^2 tests, P<0.05; *Supplementary Data 5*). **b.** Representation of Asian individuals in individual pivotal trials that led to FDA-approved drugs in 2023. X-axis: Each red column represents a trial. For 54 of the 55 drug trials the percent enrollment of Asian individuals was reported by the DTSP. Y-axis: The percent enrollment of Asian individuals is subtracted from their US Census statistics for 2023 (6.4%). Clinical trials inside the light red box show significant underrepresentation of Asian individuals (two-sided χ^2 tests, P<0.05; *Supplementary Data 5*).

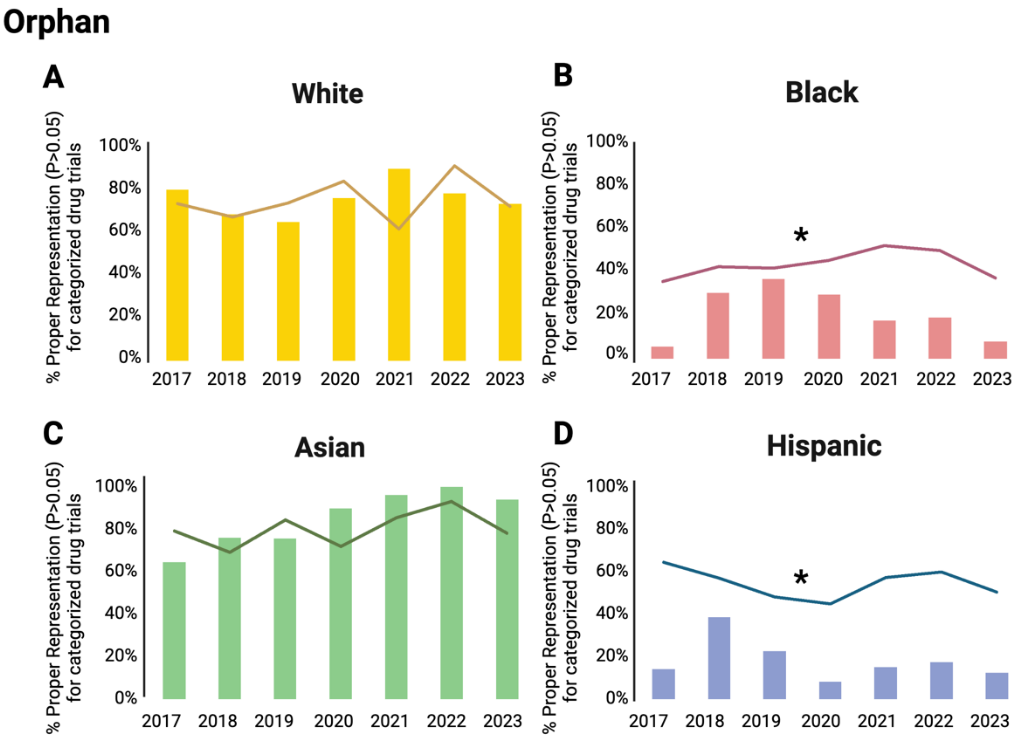

Supplementary Fig. 2 | Representation of racial and ethnic groups in clinical trials over time in relation to Orphan drug designation status. a. Representation of White individuals in clinical trials for Orphan and Non-Orphan drugs over time. X-axis: All trials for Orphan drugs for each year are represented in a single column, while all trials for Non-Orphan drugs for each year are represented in a data point on the line. Y-axis: The percent clinical trials that show appropriate representation of White individuals (two-sided χ^2 tests, P>0.05; *Supplementary Data 5*) compared to the US Census data for that year. **b.** The same analyses as in (**a**) were repeated for representation of Black individuals. The asterisk indicates a significant difference in representation of Black individuals between trials for Orphan and Non-Orphan drugs across years (Kolmogorov-Smirnov test, KS D_{Black}=0.86, P=0.008). **c.** The same analyses as in (**a**) were repeated for representation of Asian individuals. **d.** The same analyses as in (**a**) were repeated for representation of Hispanic individuals. The asterisk indicates a significant difference in representation of Hispanic individuals between trials for Orphan and Non-Orphan drugs across years (Kolmogorov-Smirnov test, KS $D_{Hispanic}$=1.00, P=0.000058). Overall, the trial sizes for Orphan drugs in 2022-2023 range from n=10-539 individuals (Mean=166), which is significantly smaller (Student's t=3.0240, P=0.0038) than the trial sizes for Non-Orphan drugs that range from 29-11,806 (Mean=1,457).

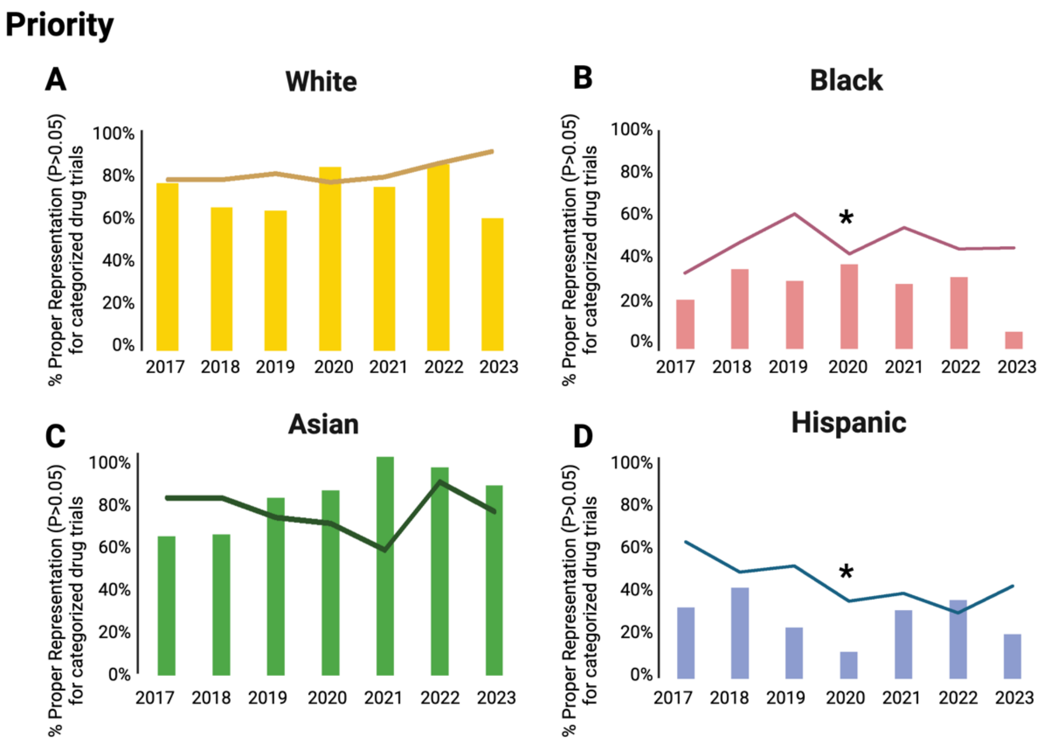

Supplementary Fig. 3 | Representation of racial and ethnic groups in clinical trials over time in relation to Priority Review drug designation status. a. Representation of White individuals in clinical trials for drugs with and without Priority designation over time. X-axis: All trials for Priority drugs for each year are represented in a single column, while all trials for Non-Priority drugs for each year are represented in a data point on the line. Y-axis: The percent clinical trials that show appropriate representation of White individuals (two-sided χ^2 tests, P>0.05; *Supplementary Data 5*), compared to the US Census data for that year. **b.** The same analyses as in (**a**) were repeated for representation of Black individuals. The asterisk indicates a significant difference in representation of Black individuals between trials for Priority and Non-Priority drugs across years (two-sided Kolmogorov-Smirnov test, P<0.05). **c.** The same analyses as in (**a**) were repeated for representation of Asian individuals. **d.** The same analyses as in (**a**) were repeated for representation of Hispanic individuals. The asterisk indicates a significant difference in representation of Hispanic individuals between trials for Priority and Non-Priority drugs across years (two-sided Kolmogorov-Smirnov test, P<0.05).

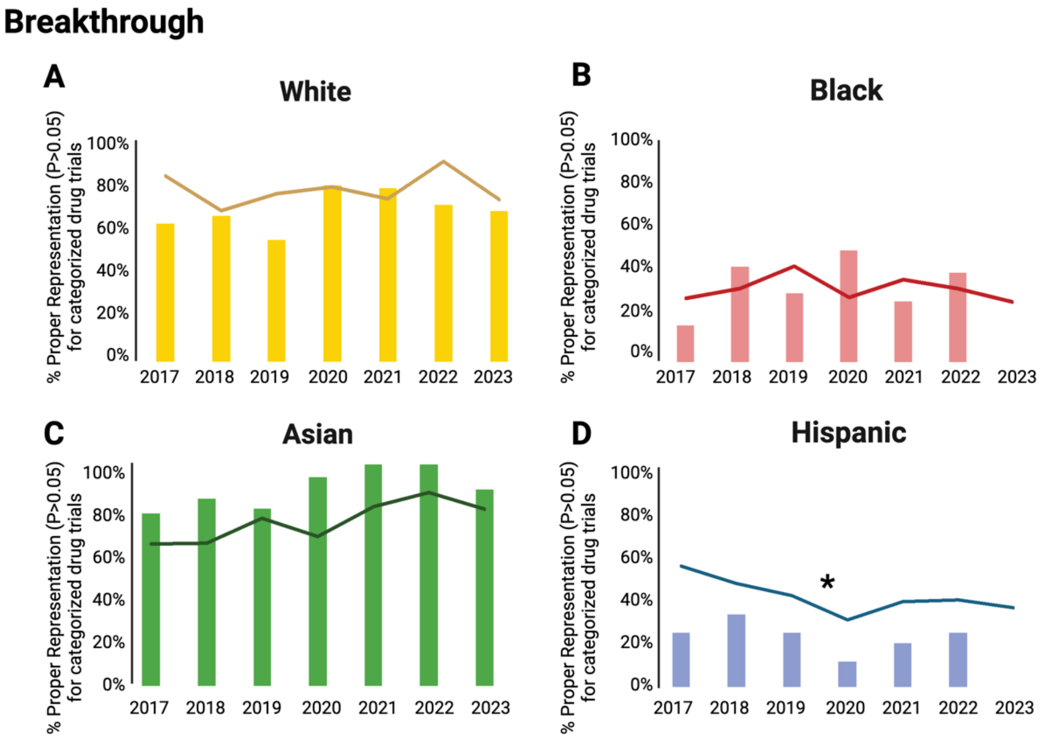

Supplementary Fig. 4 | Representation of racial and ethnic groups in clinical trials over time in relation to Breakthrough Therapy drug designation status. a. Representation of White individuals in clinical trials for drugs with and without Breakthrough designation over time. X-axis: All trials for Breakthrough drugs for each year are represented in a single column, while all trials for Non-Breakthrough drugs for each year are represented in a data point on the line. Y-axis: The percent clinical trials that show appropriate representation of White individuals (two-sided χ^2 tests, $P>0.05$; *Supplementary Data 5*), compared to the US Census data for that year. **b.** The same analyses as in (**a**) were repeated for representation of Black individuals. **c.** The same analyses as in (**a**) were repeated for representation of Asian individuals. **d.** The same analyses as in (**a**) were repeated for representation of Hispanic individuals. The asterisk indicates a significant difference in representation of Hispanic individuals between trials for drugs with and without Breakthrough designation across years (two-sided Kolmogorov-Smirnov test, KS $D_{Hispanic}=0.86$, $P=0.00483$).

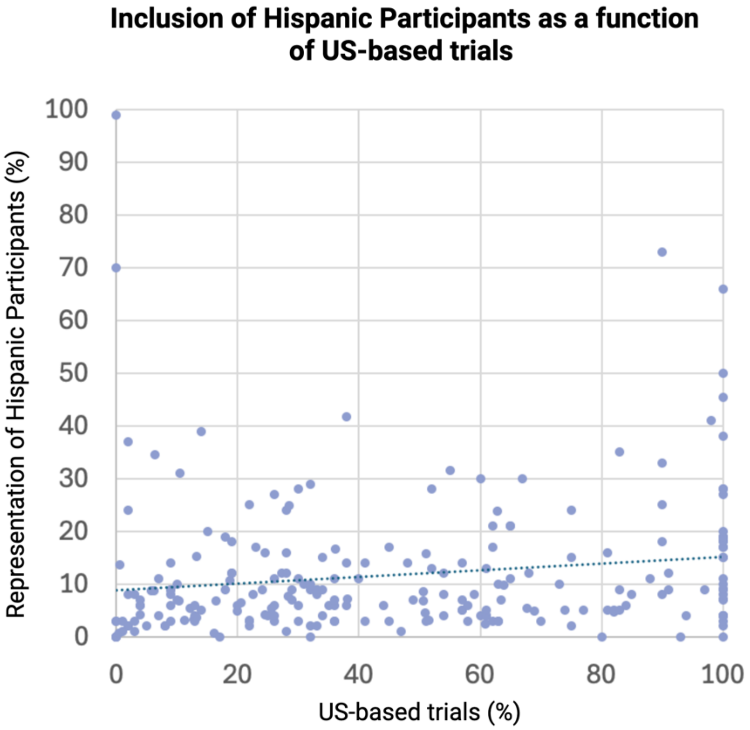

Supplementary Fig. 5 | Representation of Hispanic individuals in clinical trials over time in relation to trial location. Regression analysis of representation of Hispanic individuals in trials on the proportions of trial participants based in the US. X-axis: Each dot represents a trial. Y-axis: The percent enrollment of Hispanic individuals in each trial is shown. The dotted line indicates a marginally significant linear regression fit as determined by a two-sided F-test (F=6.05, P=0.0147).

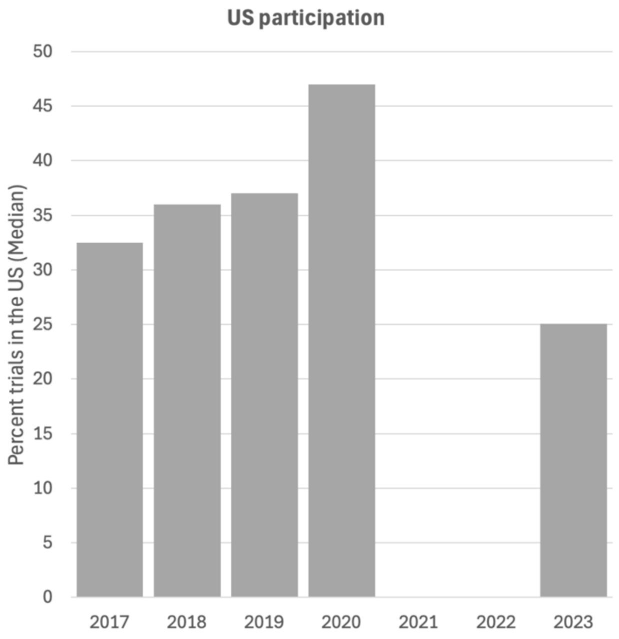

Supplementary Fig. 6 | The percentage of US-based enrollment for each clinical trial as provided by Drug Trial Snapshots Program reports. The bar plot displays the median value across all trials for each year. Note that location data was not included in the reports for 2021 and 2022, which explains the missing bars.

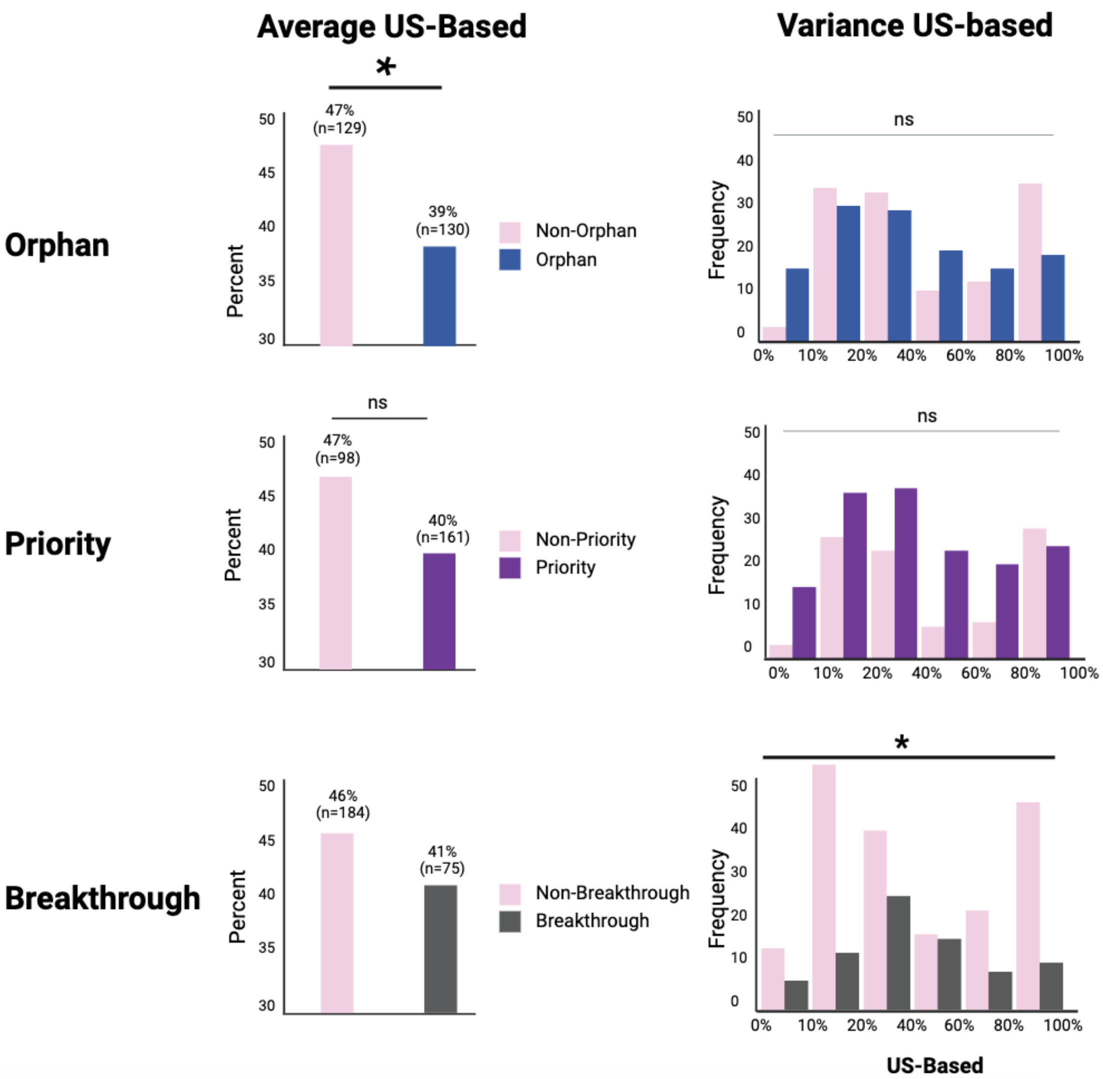

Supplementary Fig. 7 | Percent enrollment of US-based individuals in clinical trials for drugs approved in the period from 2017 to 2023 categorized by drug designation (Orphan, Priority Review, and Breakthrough Therapy). In the bar plots on the left side the average enrollment percentage of US-based individuals per trial is shown. Only trials for drugs with Orphan designation had an overall average enrollment of US-based individuals that differed from their enrollment in trials for drugs without such designation (two-sided Student's t-test, P=0.03682). The bar plots on the right side show the distribution of US-based participants in trials for drugs with each designation. Only trials for drugs with Breakthrough designation showed a different distribution (two-sided Levene's test, P=0.0037) compared to trials for drugs without such designation.

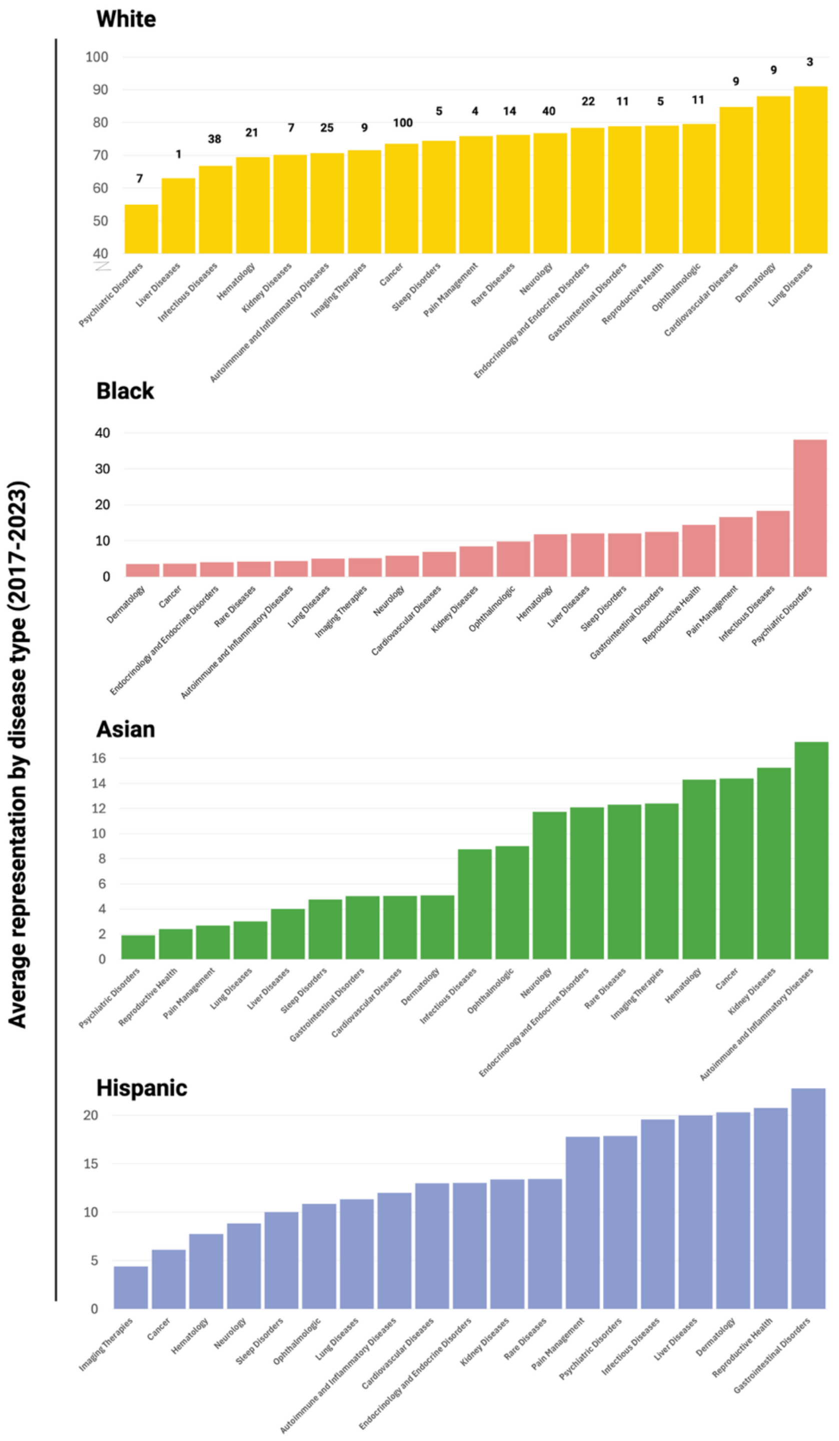

Supplementry Fig. 8 | Representation of White, Black, Asian, and Hispanic individuals in clinical trials for drugs approved between 2017 and 2023, categorized by disease. X-axis: A single column represents all trials within each disease category. Y-axis: The percentage of each demographic group enrolled across trials for each disease category is shown. The number of trials for each category is indicated in the top panel and is the same for all four demographic groups. Further information can be found in *Supplementary Data 7*.
