# Supplementary material for: Longitudinal clinical trial enrollment trends across 341 US FDA-approved drugs and their guiding role in precision medicine strategies
Source: Commun Med (Lond). 2025 Dec 5;5:514. doi: 10.1038/s43856-025-01270-2 (PMC12680696; doi:10.1038/s43856-025-01270-2)
Supplement: Supplementary file 2 — Description of Additional Supplementary files [file 43856_2025_1270_MOESM2_ESM.pdf]

Description of Additional Supplementary Files

File name: Supplementary Data 1

Description: PRISMA reporting form

File name: Supplementary Data 2

Description: Study data and metadata references

File name: Supplementary Data 3

Description: Frequency of reporting for each human demographic group in drug trial snapshot program reports

File name: Supplementary Data 4

Description: Number of demographic groups with reported information for each drug trial

File name: Supplementary Data 5

Description: Number of trials with underrepresented demographic groups separated by combinations of drug designations

File name: Supplementary Data 6

Description: Annual statistics on drug trial participation for demographic groups and on US-based participation

File name: Supplementary Data 7

Description: Statistics on drug trial participation for demographic groups grouped by disease types
